# Hoffa fracture combined with rotational dislocation of the knee joint: A novel case report

**DOI:** 10.1097/MD.0000000000025253

**Published:** 2021-04-09

**Authors:** Guanning Huang, Minglei Zhang, Youjia Zhang, Xukai Wang, Mingran Zhang, Guangyao Liu

**Affiliations:** a Department of Orthopedics; b Department of Nuclear Medicine, China-Japan Union Hospital of Jilin University, Changchun, Jilin, P.R. China.

**Keywords:** buttress plate, dislocation, femoral condyle, Hoffa fracture

## Abstract

**Rationale:**

Hoffa fracture is a rare fracture confined to the coronal-plane involving femoral condyles. This occurs simultaneously with rotational dislocation of the knee joint is extremely rare. Up to now, there is no valid recommendation for the treatment of the Hoffa fracture.

**Patient concerns:**

A 50-year-old female patient broke her knee joint while skiing, experiencing severe pain in the right knee, which was swollen. She presented limited function of the knee and movement upon arrival in the emergency room.

**Diagnosis:**

Comminuted Hoffa fracture in the right knee associated with rotational dislocation in the knee joint

**Interventions:**

We treated the dislocated knee joint through manual reduction initially. During the operation, we used posterolateral approach to expose the fracture fragments, thereafter using headless compression screws and a buttress plate to provide sufficient stability for the fracture. Early postoperative rehabilitation was encouraged.

**Outcomes:**

The patient finally achieved fracture healing three months after operation. In addition, she achieved 0–130° range of function of the knee after four months post-operation, and the patient obtained a satisfactory prognosis after our treatment.

**Lessons:**

By using appropriate surgical approach to obtain enough exposure, headless compression screws and the buttress plate provided adequate stability during early active rehabilitation, which resulted in satisfactory results in the treatment of the injury. We reviewed literatures regarding the treatment of Hoffa fracture to demonstrate that our treatment was effective.

## 1 Introduction

Hoffa fracture is a very rare fracture, the incidence of the fracture merely accounts for 0.65% of all fractures.^[1]^ Many literature sources reported that this type of distal femoral coronal plane fracture was first discovered by Hoffa. However, some scholars believed that Busch was the first one who described the type of fracture in 1869.^[2]^ Hoffa fracture is mainly caused by high-energy injuries. The mechanism of this injury is that during knee flexion, force is transmitted from the distal end of the limb to distal end of the femur, and the most common condition is injury caused by traffic accidents.^[3]^ Additionally, Hoffa fracture is also caused by violent rotation of the knee joint.^[4]^ According to relative literature sources, the incidence of fracture of lateral condyle is significantly higher than the medial condyle, which may be related to the physiological valgus of the knee joint.^[5,6]^ Hoffa fractures are often associated with supracondylar and intercondylar fractures of the femur, and are accompanied with high rates of missed diagnosis on the plain radiograph. Therefore, when we find supracondylar or intercondylar fractures of the femur on the X-ray, we should consider whether these include Hoffa fractures.^[5]^ The use of computerized tomography (CT) has been recommended for patients with highly suspected Hoffa fracture or no obvious displacement of fracture.^[7]^ Hoffa fractures are intra-articular fracture, and thus the principle of treatment is the same as other fractures involving articular surface. Anatomical reduction, rigid fixation and early functional rehabilitation should be achieved to obtain better postoperative knee function. Letenneur divided Hoffa fracture into three types according to the position of the fracture line. From there, he specified type II into three different subtypes. The classification mainly emphasized the relationship between the morphology of fracture fragments and blood supply. Compared with type I and type III, the fracture of type II has smaller fragment sizes, fewer blood supply and higher probability of nonunion after surgery.^[8]^ The option of surgical approach should consider the morphology, size and position of the fracture fragments. An appropriate approach is not only to obtain adequate exposure, but to also avoid excessive dissection of the soft tissue which would disrupt the blood supply. Internal implants mainly involve using cannulated screws or locking buttress plates. In recent years, some scholars have suggested that cannulated screws combined with an anti-sliding plate should be used to immobilize the fracture, as more rigid fixation contributed to receive better postoperative knee function.^[9–11]^ Due to the diversity of surgical approaches and internal fixation methods, currently there is no consensus on the optimal treatment for Hoffa fracture. The incidence of Hoffa fracture combined with dislocation of knee joint is extremely rare. At the point of injury, the popliteal artery, collateral ligaments, cruciate ligaments and the meniscus may be injured. The high-energy injury may result in poor knee function after surgery. In our report, we present a comminuted fracture of lateral femoral condyle with dislocation of knee joint associated with bilateral collateral ligaments, anteroposterior cruciate ligaments rupture and lateral meniscus tear. To the current author's knowledge, this is the first case report regarding lateral Hoffa fracture and anterolateral dislocation in knee joint. In addition, we also provide a review of previous literatures.

## 2 Case presentation

**Figure 1:**
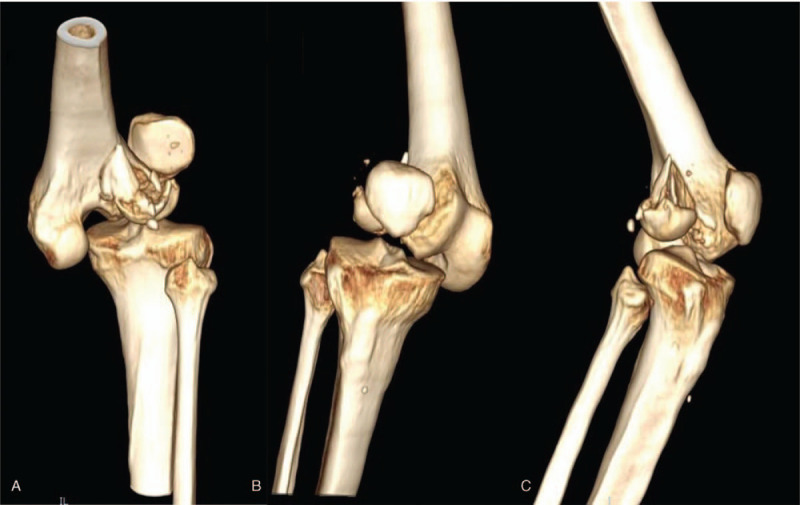
(A–C) The CT of knee joint showed the comminuted lateral Hoffa fracture combined with rotational dislocation of the knee joint.

**Figure 2:**
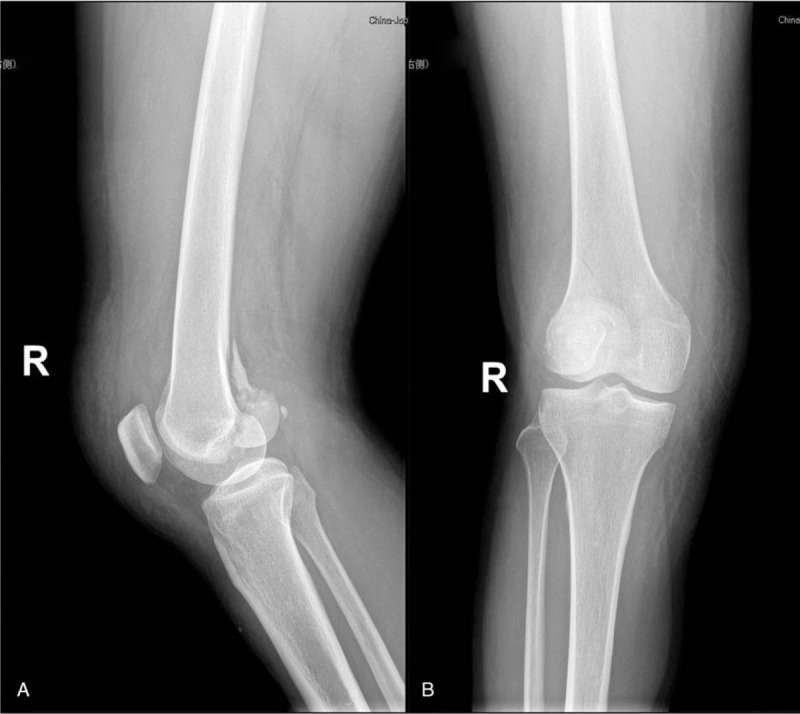
(A and B) AP view and lateral view demonstrated the dislocation of knee joint has been restored.

**Figure 3:**
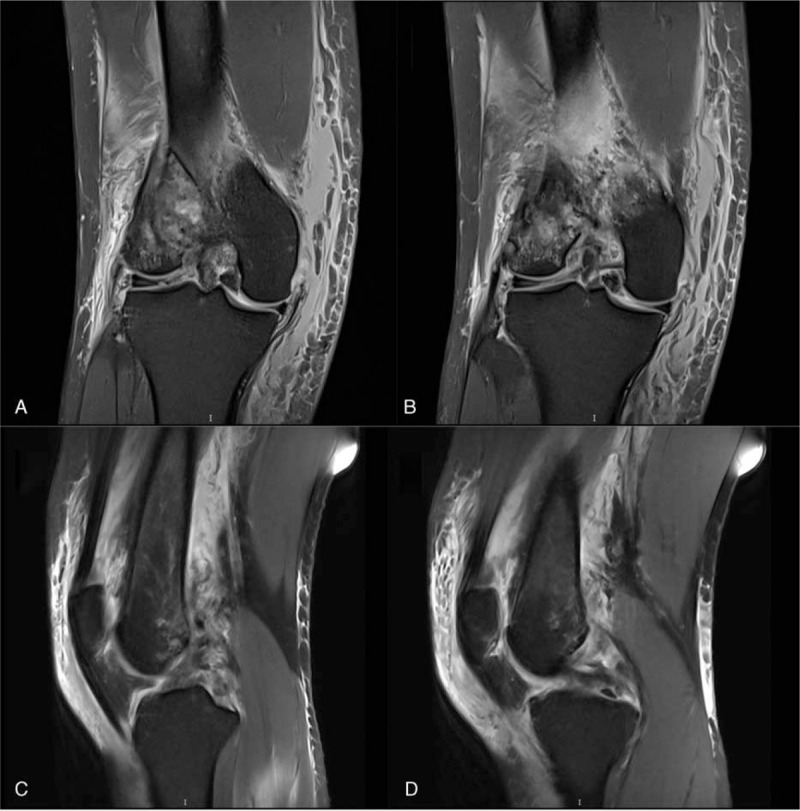
(A–D) The preoperative MRI images of the knee clearly showed medial and lateral collateral ligaments, anterior and posterior cruciate ligaments suffered varying degrees of injury.

**Figure 4:**
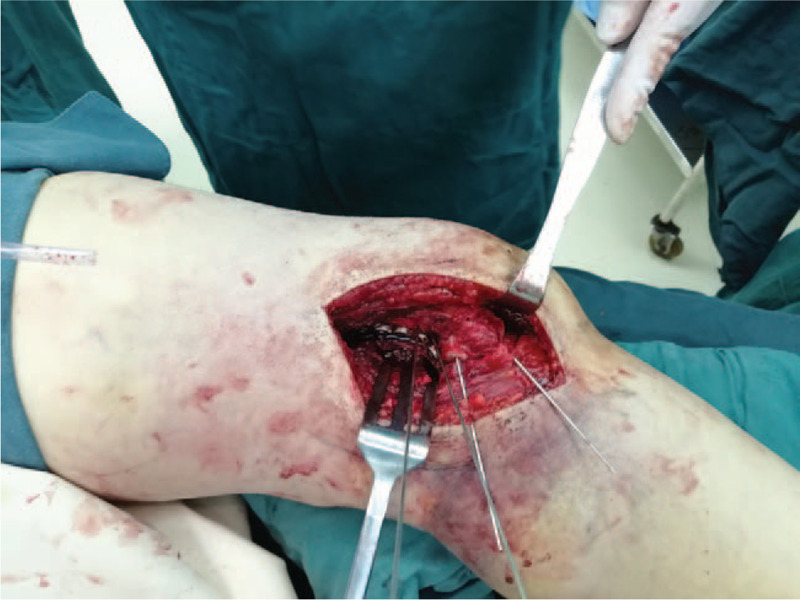
Intraoperative imaging showed that we provided two 4.5 mm headless compression screws and placed them perpendicular to fracture line from posterior-anterior direction, a locking plate was anatomically contoured and placed it behind the lateral condyle.

**Figure 5:**
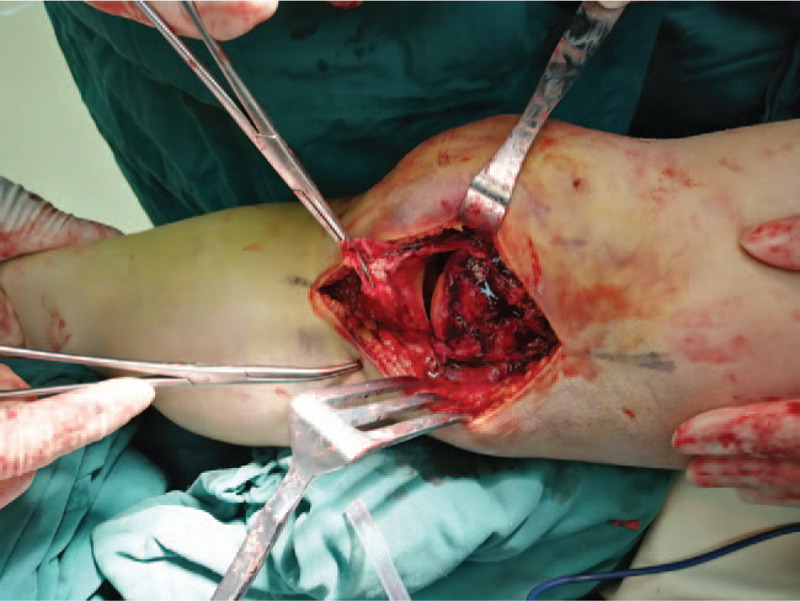
Intraoperative imaging demonstrated complete rapture of the medial collateral ligament.

**Figure 6:**
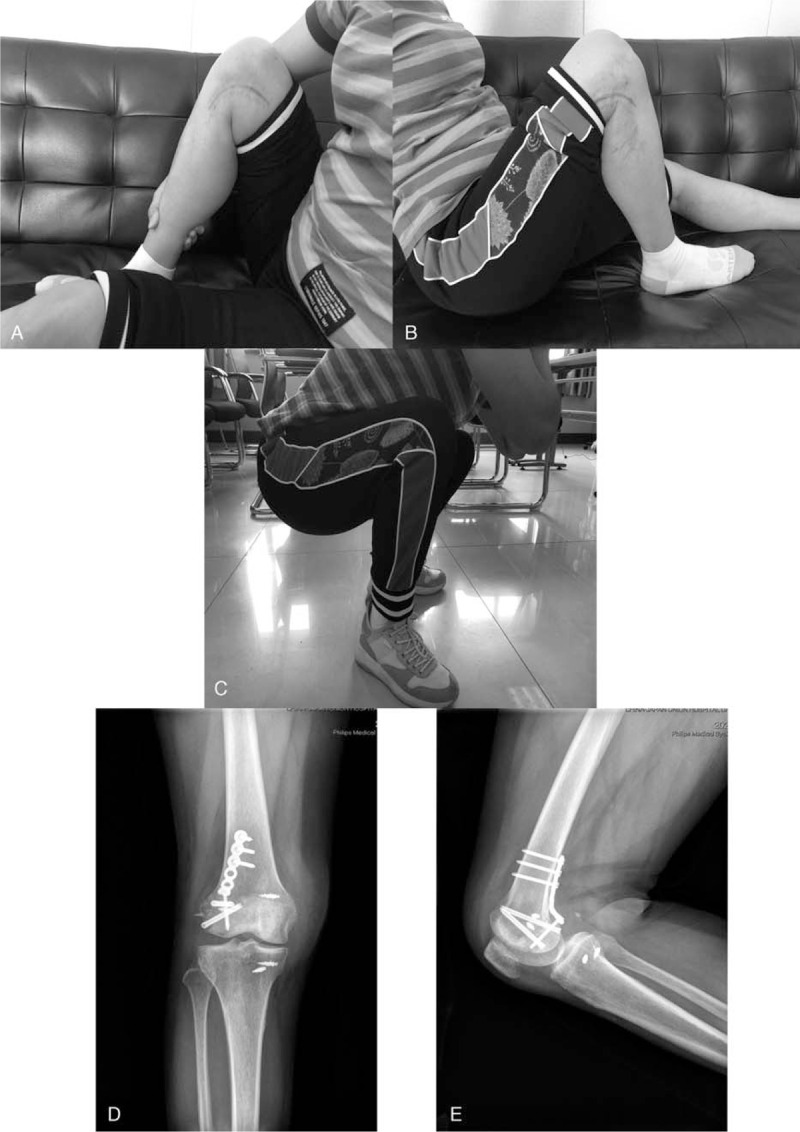
(A, B, C) After four months, the range of motion of the knee has achieved 0–130°. (D, E) AP view and lateral view showed the fracture line disappeared on the radiograph and achieved fracture healing.

A 50-year-old female patient, who broke her knee joint while skiing. When she arrived at the emergency room, she experienced severe pain in the right knee, which was swollen and had functional limitation. Lateral radiograph and CT better revealed comminuted fracture of the distal posterolateral femoral condyle and rotational dislocation of the knee joint (Fig. 1). Physical examination showed the pulse of dorsalis pedis arteria and posterior tibial artery could be detected, with no abnormal sensation over the affected limb. The dorsal and plantar flexion function of the ankle joint was normal. In order to avoid the damage of peripheral vessels and nerves caused by dislocation of the knee joint, we first applied manual reduction on the dislocated knee joint (Fig. 2). Furthermore, MRI results clearly reported a complete rupture of bilateral collateral ligaments, anteroposterior cruciate ligaments and avulsion of lateral meniscus (Fig. 3). We did not perform the Lachman tests and Bohler sign due to the drastic pain in the right knee. In order to prevent arthrofibrosis of knee joint caused by hematoma in the future, the patient underwent knee joint puncture under ultrasound guidance and had 100 ml of bloody fluid drained. Then, we used elastic bandages to compress the affected knee joint in order to prevent the formation of hematoma. After five days, the swelling subsided and we were able to operate. Initially, the patient was placed in the floating position with the affected limb elevated. Preoperative antibiotics and general anesthetic were administered. Then a posterolateral approach was adopted, we went through the interval space between the biceps and the common peroneal nerve, the popliteus and the lateral head of the gastrocnemius were partial dissected with caution. A horizontal capsulotomy is performed to expose the posterior femoral condyle and allow P-A screw fixation perpendicular to the fracture line. Several kirschner wires were used to temporarily immobilize the fragment fracture after it was reduced by the pointed reduction clamp, then took two 4.5 mm headless compression screws with full thread and placed them perpendicular to fracture line from posterior- anterior orientation. In addition, a locking plate was anatomically contoured and placed it behind the lateral condyle (Fig. 4). The anti-slip plate can better resist shear force and provide angular stability, and also prevent the vertical gliding of the fragment. During the operation, we found the lateral meniscus wedged inside the tibiofemoral articular surface, and the lateral collateral ligament was ruptured at the insertion of the femur. Then we released the injured meniscus and immobilized it to the lateral capsule, the anchor suture was used to reconstruct the insertion of the LCL. Thereafter, the patient was placed in the supine position and we took the direct medial approach through the intermuscular plane between the gracilis and semimembranosus. We discovered a complete rapture of the medial collateral ligament (Fig. 5) and anchor sutures were used to reestablish both ends of the breakage. After repairing the bilateral collateral ligaments, we checked the stability of the knee joint during intraoperative surgery, and the bohler sign was negative. However, the lachman test was positive. We decided to repair the cruciate ligaments during the second stage of surgery after fracture healing. Rehabilitation training started immediately after surgery and continued for four months. The patient achieved 0–90° range of function of knee after active rehabilitative exercise two weeks after operation. Thereafter, 0–130° range of function was achieved after four months, and the fracture line disappeared on the radiograph results. Moreover, the patient was walking with no limitation and was able to move without using walking aids (Fig. 6), the Knee Society Scores (KSS) of the patient was reached 80. After six months, we eventually removed the internal fixations and reconstructed the cruciate ligaments under the arthroscopy.

## 3 Discussion

To our best knowledge, this is the first case report of Hoffa fracture associated with anterolateral dislocation of the knee. Hoffa fracture is an uncommon fracture, and the combination of the dislocation of knee is an even rarer occurrence. Hoffa fracture is also an intra-articular fracture, and conservative treatment in the past often resulted in secondary displacement of fracture fragment and worse knee function.^[8,12]^ Some scholars suggested the principle of treatment of Hoffa fracture is anatomical reduction of articular surface, preservation of the blood supply of the fracture, rigid internal fixation, early aggressive rehabilitation. However, the treatment of Hoffa fracture still remains controversial and challenging due to variation of surgical approaches, fixation methods and functional exercise. Hoffa fracture is easily missed on the X-ray, particularly as it has no obvious displacement. This is also the case with intercondylar or supracondylar fractures. Therefore, in order to avoid missing the fracture and better understand the morphology of fracture, the use of computerized tomography (CT) has been recommended.^[5,8,13]^ In 1978, Letenneur proposed the classification of the Hoffa fracture into three types. A type I fracture line is parallel to the posterior femoral cortex and involves the intact posterior condyle. A type II fracture is still a fracture line parallel to the posterior femoral cortex, but the fracture line is posterior to type I. Type II fracture is subdivided into A, B and C according to fragment size based on percentage of condyle diameter. Type III is an oblique fracture of posterior condyle. The main significance of this classification is to predict the risk of femoral condyle avascular necrosis rather than to help us further understand the morphology of fracture. Xie et al^[14]^ reported the fracture line direction and the common area of comminuted fracture of 75 patients with Hoffa fracture by three-dimensional CT mapping techniques. They found that in axial view of the lateral femoral condyle, the trajectory of fracture lines was usually from anterolateral to posteromedial. In the medial condyle, the trajectory of fracture lines was commonly extending from anteromedial to posterolateral. Additional, in sagittal plane, whether in the lateral or medial condyle, the majority of fracture lines was running from anteroinferior to posterosuperior direction. Moreover, comminuted areas were discovered to be concentrated in the middle-third and weight-bearing area of the condyle. We can make a more meticulous preoperative plan by improving the understanding of the morphology of Hoffa fractures. Undoubtedly, this study has guiding significance to the choice of surgical approach and internal fixation method. When faced with a patient with dislocation of the knee joint, the major concern should be neurovascular injury and following is the sequelae of soft tissue disruption. Injury of ligamentous structures of knee does not only result in instability but also restrict the range of knee function. Schenck et al^[15]^ reported a case of medial Hoffa fracture with dislocation of knee joint. They provided the patient open reduction and internal fixation of fracture, and reconstructed the injured posterior cruciate ligament. However, in spite of early rehabilitation training postoperatively is performed, the patient still developed knee stiffness. Finally, he received an arthroscopic synoviectomy. Shetty et al^[16]^ reported an irreducible knee dislocation associated with Hoffa fracture. Preoperative MRI of the affected knee showed the patellar tendon has been avulsed from the inferior patella and incarcerated in the fracture line of the lateral femoral condyle. In addition, the anterior and posterior cruciate ligaments were torn at attachments respectively. After open reduction and internal fixation of the fracture in the first stage, the author intended to reconstruct the cruciate ligaments under arthroscopy after fracture healing. However, three months after operation, the patient started complaining that the range of motion of knee joint was obviously limited and the flexion could only be achieved to 0–80° motion. At this point, with no distinct instability of knee or ligaments laxity, X-ray of the affected knee demonstrated union of fracture. Then an arthroscopic arthrolysis was performed, but there was no significant improvement of the range of motion post-operation. Up to now, there are few literatures regarding the choice of surgical approaches and internal fixation methods for Hoffa fracture. Xie et al^[14]^ reported that Hoffa fracture commonly occurred in the lateral condyle. Compared with the lateral condyle, fracture lines were less concentrated in the medial condyle and comminution zones mainly distributed in the middle-third region. Consequently, the medial parapatellar approach is still the standard approach for the most medial Hoffa fracture. Viskontas et al recommend to use medial subvastus approach to treat medial Hoffa fracture, the approach was initially used for total knee arthroplasty.^[17]^ Compared with the traditional medial parapatellar approach, the approach can better protect the knee extensor and patellar blood supply.^[18,19]^ Moreover, the anterior and posterior regions of the medial femoral condyle can be greater exposed through two surgical window. Gao et al^[9]^ reported that used posteromedial approach to treat medial Hoffa fracture, they exposed the fracture line adequately through the interval space between the gracilis and the medial head of the gastrocnemius. The approach is appropriate for the Hoffa fracture in cases where the fracture line extends to the metaphysis which requires an extra medial locking plate. Recently, Orapiriyapul et al^[20]^ have done a lot of work regarding the option of surgical approach for Hoffa fracture. They concluded that the traditional parapatellar approach was insufficient to expose comminuted fragment for Letenneur IIb and IIc Hoffa fracture. If the fracture fragment is very small, while less than 28.7% of the diameter of the medial femoral condyle and the fracture line does not extend to metaphysis, the direct medial approach is recommended. Compared to parapatellar approach, the advantages of this approach include not damaging any knee structure and better expose the posterior aspect of medial condyle. Lian et al^[10]^ recommended to selecting the posterolateral approach to treat lateral Hoffa fracture, the fracture line and comminuted area can be adequately exposed to achieve rigid fixation of fracture with buttress plate and cannulated screws. In addition, all the fracture healed without complications in 12 patients. Orapiriyakul et al^[21]^ also preferred to use posterolateral approach for lateral Hoffa fracture if the fracture fragment is less than 19.9% of the diameter of the lateral condyle. This approach allows direct visualization of the fracture and achieves perpendicular screw fixation of the fracture line in the posterior-to-anterior direction. The option of which fixation method can provide stronger fixation for Hoffa fracture is also controversial. Only if the fixation strength achieves biomechanical stability, patients are allowed to early aggressive rehabilitation post-operation without protection, which is crucial for the knee function. Kumar and Malhotra^[22]^ reported three cases of Hoffa fracture were treated with anterior to posterior direction screws, and all patients obtained satisfactory results. Holmes et al.^[23]^ reported five patients treated with anterior to posterior direction cancellous lag screws, in one case, nonunion occurred, while in the other cases, fracture healed within three months. Jarit et al^[24]^ discovered that the use of two 6.5 mm partially threaded cancellous lag screws with posterior to anterior direction was more stable than the anterior to posterior screws for the first-time in vitro study. The displacement of fracture was lesser when provided the same load. Furthermore, the fixation method had a higher maximal strength to resist external force. However, P-A direction screws often required additional countersunk head, which prevents damage to the articular cartilage. Hak et al^[25]^ reported the biomechanical analysis of four different screw fixation methods. They found that the firmness of two 6.5 mm screws was significantly better than the use of two 3.5 mm screws and one 3.5 mm screw. There was no statistical difference in stability between the use of two 6.5 mm screws and the single 6.5 mm screw. However, if 3.5 mm screws are used, at least two screws are required to achieve the biomechanical stability of the single 6.5 mm screw. Some scholars believed that the ideal fixation method for Hoffa fracture is to use the minimum diameter and minimum number of screws to achieve sufficient rigid fixation, with minimal damage to the articular cartilage. Subsequently, Borse et al^[26]^ firstly used two headless compression screws in posterior to anterior direction in clinical cases. They considered that using headless compression screws in posterior to anterior direction and perpendicular to the fracture line did not only efficiently resisted shear and rotational forces but also minimized the damage to the articular surface. With the increase of the traffic accidents and aging population, high-energy comminuted Hoffa fracture or osteoporotic cases provides a challenge to orthopedists. In such situation, the use of screws alone may not provide effective fixation. In this case, providing a buttress plate seems to improve the clinical outcomes, prevent secondary displacement of fracture and even failure of internal fixation. Gao et al^[9]^ reviewed the previous literature sources and found that it is difficult to achieve enough stable fixation of Hoffa fracture with single plate or screws. They reported 13 cases of medial Hoffa fracture were treated with posterior medial buttress plate associated with PA direction screws. All patients achieved bone healing and restored the initial mobility through active postoperative rehabilitation training. Moreover, the Knee Society Scores (KSS) of all the patients were more than 80. Sun et al^[11]^ performed a biomechanical analysis of four different fixation methods by simulating a Letenneur I Hoffa fracture. They found that the control group of a 6.5 mm PA direction lag screws combined with a lateral buttress plate provided the strongest fixation and minimal displacement of fracture. In addition, the stability of the group of two 6.5 mm AP direction lag screws was worst, provided minimal fixation strength and maximal vertical displacement. Lu et al^[27]^ reported a retrospective study of 47 Hoffa fractures. All patients were divided into the group of screws combined with plate and single screws. All cases achieved bone healing, however, the range of movement of knee and Knee Society Scores in the group which had screws combined with plate were better than the single-screws group in the postoperative follow-up. The authors believed that the plate may provide sufficient stability to achieve early aggressive rehabilitation and result in better functional outcomes. To our knowledge, most scholars advocate early postoperative functional training. Gavaskar et al^[28]^ reported 18 patients of Hoffa fracture who received satisfactory knee function through early active rehabilitation. Gao et al^[9]^ advocated early passive training of the affected knee by CPM system after the relief of edema and pain. In general, three weeks after operation, the patients achieved a range 0–130 knee function with little or no pain. After ten weeks, the patients were able to handle partial weight-bearing with crutches. When the signs of bone healing were revealed on the X-ray, the patients were allowed to handle full weight-bearing. However, the starting point of rehabilitation varies from person to person, and individualized treatment should be performed. It is necessary to take into account the method and strength of the internal implants. Otherwise, aggressive exercises may lead to secondary displacement of the fracture and even failure of the internal fixation.

In conclusion, to our best knowledge, this is the first English case report of Hoffa fracture associated with anterolateral dislocation in the knee. Literature sources are lacking regarding such case due to extremely low incidence of the injury, the treatment guidelines in the situation are still unclear. In general, the postoperative function of such comminuted Hoffa fracture combined with multiple ligamentous injuries is much worse when compared to either injury alone. However, we achieved enough exposure of the fracture through reasonable surgical approach, and the use of headless compression screws combined with buttress plate, to provide adequate stability, and we advocated early active rehabilitation to obtain a satisfactory function of knee. Undoubtedly, the treatment we have used in this case is effective, but we still need more clinical studies to better guide us in treating this injury in future.

## 4 Patient consent

Written informed consent was acquired from the patient for publication of the case report. All information about the patient in the manuscript complied with the right of the patient and is authorized by the patient. The ethical approval was provided by the Ethics Committee of the China-Japan Union Hospital of Jilin University.

## Author contributions

**Conceptualization:** Guanning Huang, Guangyao Liu.

**Data curation:** Guanning Huang, Minglei Zhang, Youjia Zhang, Xukai Wang, Mingran Zhang.

**Formal analysis:** Guanning Huang, Youjia Zhang.

**Investigation:** Guanning Huang, Minglei Zhang, Mingran Zhang, Guangyao Liu.

**Methodology:** Xukai Wang, Mingran Zhang.

**Project administration:** Guangyao Liu.

**Resources:** Guanning Huang, Mingran Zhang.

**Software:** Guanning Huang, Minglei Zhang, Youjia Zhang, Xukai Wang.

**Supervision:** Guangyao Liu.

**Visualization:** Minglei Zhang, Youjia Zhang.

**Writing – original draft:** Guanning Huang, Minglei Zhang, Guangyao Liu.

**Writing – review & editing:** Guanning Huang, Guangyao Liu.
